# 6,6-Dimethyl-2*H*,5*H*,6*H*,7*H*-1,3-dithiolo[4,5-*f*][1,5,3]dithia­silepin-2-one

**DOI:** 10.1107/S1600536812011142

**Published:** 2012-03-21

**Authors:** Hongqi Li, Xuebin Zhang, Zhongbao Zhang, Zhen Chen, Jiajian Peng

**Affiliations:** a Key Laboratory of Science & Technology of Eco-Textiles, Ministry of Education, College of Chemistry, Chemical Engineering & Biotechnology, Donghua University, Shanghai 201620, People’s Republic of China; b Key Laboratory of Organosilicon Chemistry and Material Technology of the Ministry of Education, Hangzhou Normal University, Hangzhou 310012, People’s Republic of China

## Abstract

In the structure of the title compound, C_7_H_10_OS_4_Si, the carbonyl O atom lies in the plane of the five-membered dithiole ring with a deviation of only 0.022 (2) Å. The seven-membered ring adopts a chair conformation. The crystal packing is stabilized by S⋯O [3.096 (4) Å] and S⋯S [3.620 (4) Å] contacts, together with C—H⋯S inter­actions.

## Related literature

For silicon-containing tetra­thia­fulvalene (TTF) derivatives as ligands, see: Guyon *et al.* (2005 ▶), and as precursors for the construction of polymetallic arrays, see: Hameau *et al.* (2008 ▶). For their use in the preparation of conducting charge-transfer complexes and radical-cation salts, see: Biaso *et al.* (2007 ▶). For the synthesis, see: Li *et al.* (2012 ▶). For related structures, see: Arumugam *et al.* (2011 ▶); Hou *et al.* (2009 ▶).
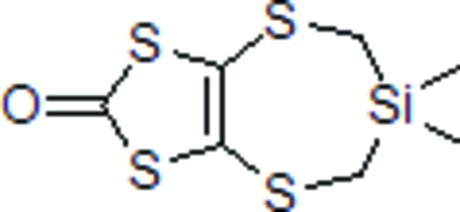


## Experimental

### 

#### Crystal data

C_7_H_10_OS_4_Si
*M* *_r_* = 266.48
Triclinic, 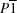

*a* = 6.148 (7) Å
*b* = 8.569 (10) Å
*c* = 11.846 (14) Å
α = 69.292 (12)°
β = 85.821 (13)°
γ = 83.129 (13)°
*V* = 579.2 (12) Å^3^
*Z* = 2
Mo *K*α radiation
μ = 0.88 mm^−1^
*T* = 296 K
0.12 × 0.10 × 0.08 mm

#### Data collection

Bruker APEXII CCD diffractometer
Absorption correction: multi-scan (*SADABS*; Sheldrick, 2004 ▶) *T*_min_ = 0.901, *T*_max_ = 0.933
4000 measured reflections
2012 independent reflections
1628 reflections with *I* > 2σ(*I*)
*R*_int_ = 0.019

#### Refinement

*R*[*F* ^2^ > 2σ(*F* ^2^)] = 0.031
*wR*(*F* ^2^) = 0.072
*S* = 1.01
2012 reflections
120 parameters
H-atom parameters constrained
Δρ_max_ = 0.25 e Å^−3^
Δρ_min_ = −0.26 e Å^−3^

### 

Data collection: *APEX2* (Bruker, 2004 ▶); cell refinement: *SAINT* (Bruker, 2004 ▶); data reduction: *SAINT*; program(s) used to solve structure: *SHELXS97* (Sheldrick, 2008 ▶); program(s) used to refine structure: *SHELXL97* (Sheldrick, 2008 ▶); molecular graphics: *SHELXTL*; software used to prepare material for publication: *SHELXTL*.

## Supplementary Material

Crystal structure: contains datablock(s) global, I. DOI: 10.1107/S1600536812011142/sj5206sup1.cif

Structure factors: contains datablock(s) I. DOI: 10.1107/S1600536812011142/sj5206Isup2.hkl

Supplementary material file. DOI: 10.1107/S1600536812011142/sj5206Isup3.cml

Additional supplementary materials:  crystallographic information; 3D view; checkCIF report

## Figures and Tables

**Table 1:** Hydrogen-bond geometry (Å, °)

*D*—H⋯*A*	*D*—H	H⋯*A*	*D*⋯*A*	*D*—H⋯*A*
C5—H5*B*⋯S1^i^	0.97	2.89	3.673 (5)	138

Symmetry code: (i) 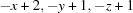.

**Fig. 1.**
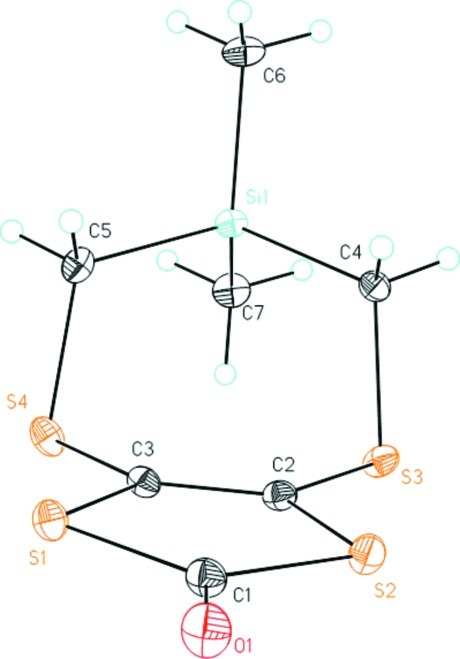
A view of the molecule of the title compound. Displacement ellipsoids are drawn at the 30% probability level and H atoms are shown as small spheres of arbitrary radii.

**Fig. 2.**
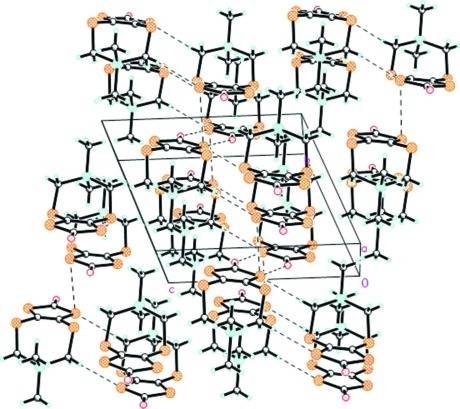
The crystal structure of the title compound, viewed along a axis

## supplementary crystallographic information

### Comment

Silicon-containing tetrathiafulvalene (TTF) derivatives have been synthesized and used as novel assembling ligands for the construction of bimetallic transition metal complexes (Guyon *et al.*, 2005), as very promising precursors for the construction of polymetallic arrays (Hameau *et al.*, 2008), or for the preparation of conducting charge transfer complexes and radical-cation salts (Biaso *et al.*, 2007). 4,5-(2,2-Dimethyl-2-silapropylene)dithio-1,3-dithiole-2-one is useful in the synthesis of new silyl-substituted TTF derivatives. Its single crystal structure has not been reported yet, though crystal structures of analogous 1,3-dithiole-2-one and 1,3-dithiole-2-thione compounds have been studied (Arumugam *et al.*, 2011; Hou *et al.*, 2009). Herein we present the single crystal structure of the title compound.

In the title compound the carbonyl-oxygen atom (O1) lies in the plane of the five-membered dithiole ring (C1-C3/S1/S2/O1) with a deviation of only -0.022 (2)Å. The seven-membered ring adopts a chair conformation. Crystal packing is characterized by intermolecular S···O interaction with S1···O1 distance of 3.096 (4)Å and S···S contacts at 3.620 (4)Å. In addition, short intermolecular C—H···.S contacts are also observed (Table 1).

### Experimental

The title compound was prepared as reported in the literature (Li *et al.*, 2012). Single crystals suitable for X-ray diffraction measurement was obtained by slow evaporation from a solution of petroleum ether and ethyl acetate (1:1).

### Refinement

All H atoms were placed at calculated positions and refined using a riding model approximation, with C—H = 0.96 or 0.97 Å and with *U*_iso_(H) = 1.2*U*_eq_(C) or 1.5*U*_eq_(C) for methyl H atoms.

### Crystal data

**Table:**

C_7_H_10_OS_4_Si	*Z* = 2
*M**_r_* = 266.48	*F*(000) = 276
Triclinic, *P*1	*D*_x_ = 1.528 Mg m^−^^3^
Hall symbol: -P 1	Melting point = 325–326 K
*a* = 6.148 (7) Å	Mo *K*α radiation, λ = 0.71073 Å
*b* = 8.569 (10) Å	Cell parameters from 1863 reflections
*c* = 11.846 (14) Å	θ = 2.6–27.4°
α = 69.292 (12)°	µ = 0.88 mm^−^^1^
β = 85.821 (13)°	*T* = 296 K
γ = 83.129 (13)°	Block, colorless
*V* = 579.2 (12) Å^3^	0.12 × 0.10 × 0.08 mm

### Data collection

**Table:**

Bruker APEXII CCD diffractometer	2012 independent reflections
Radiation source: fine-focus sealed tube	1628 reflections with *I* > 2σ(*I*)
Graphite monochromator	*R*_int_ = 0.019
φ and ω scans	θ_max_ = 25.0°, θ_min_ = 2.6°
Absorption correction: multi-scan (*SADABS*; Sheldrick, 2004)	*h* = −7→7
*T*_min_ = 0.901, *T*_max_ = 0.933	*k* = −10→10
4000 measured reflections	*l* = −14→12

### Refinement

**Table:**

Refinement on *F*^2^	Primary atom site location: structure-invariant direct methods
Least-squares matrix: full	Secondary atom site location: difference Fourier map
*R*[*F*^2^ > 2σ(*F*^2^)] = 0.031	Hydrogen site location: inferred from neighbouring sites
*wR*(*F*^2^) = 0.072	H-atom parameters constrained
*S* = 1.01	*w* = 1/[σ^2^(*F*_o_^2^) + (0.027*P*)^2^ + 0.3021*P*] where *P* = (*F*_o_^2^ + 2*F*_c_^2^)/3
2012 reflections	(Δ/σ)_max_ = 0.001
120 parameters	Δρ_max_ = 0.25 e Å^−^^3^
0 restraints	Δρ_min_ = −0.26 e Å^−^^3^

### Special details

**Table:**

Geometry. All e.s.d.'s (except the e.s.d. in the dihedral angle between two l.s. planes) are estimated using the full covariance matrix. The cell e.s.d.'s are taken into account individually in the estimation of e.s.d.'s in distances, angles and torsion angles; correlations between e.s.d.'s in cell parameters are only used when they are defined by crystal symmetry. An approximate (isotropic) treatment of cell e.s.d.'s is used for estimating e.s.d.'s involving l.s. planes.
Refinement. Refinement of *F*^2^ against ALL reflections. The weighted *R*-factor *wR* and goodness of fit *S* are based on *F*^2^, conventional *R*-factors *R* are based on *F*, with *F* set to zero for negative *F*^2^. The threshold expression of *F*^2^ > σ(*F*^2^) is used only for calculating *R*-factors(gt) *etc*. and is not relevant to the choice of reflections for refinement. *R*-factors based on *F*^2^ are statistically about twice as large as those based on *F*, and *R*- factors based on ALL data will be even larger.

### Fractional atomic coordinates and isotropic or equivalent isotropic displacement parameters (Å^2^)

**Table:**

	*x*	*y*	*z*	*U*_iso_*/*U*_eq_	
C1	1.2928 (4)	0.0788 (3)	0.3530 (2)	0.0504 (7)	
C2	0.9211 (4)	0.2483 (3)	0.2614 (2)	0.0417 (6)	
C3	0.9299 (4)	0.2691 (3)	0.3675 (2)	0.0406 (6)	
C4	0.7687 (4)	0.5515 (3)	0.0965 (2)	0.0439 (6)	
H4A	0.7085	0.6027	0.0167	0.053*	
H4B	0.9260	0.5568	0.0873	0.053*	
C5	0.7677 (4)	0.5943 (3)	0.3452 (2)	0.0476 (7)	
H5A	0.9237	0.6060	0.3372	0.057*	
H5B	0.7013	0.6636	0.3904	0.057*	
C6	0.7323 (5)	0.8941 (3)	0.1163 (3)	0.0650 (8)	
H6A	0.6818	0.9381	0.0351	0.097*	
H6B	0.8890	0.8919	0.1147	0.097*	
H6C	0.6672	0.9640	0.1601	0.097*	
C7	0.3523 (4)	0.6708 (4)	0.2051 (3)	0.0597 (8)	
H7A	0.2889	0.7372	0.2519	0.090*	
H7B	0.3200	0.5568	0.2443	0.090*	
H7C	0.2920	0.7145	0.1261	0.090*	
O1	1.4633 (3)	−0.0091 (3)	0.37000 (19)	0.0718 (6)	
S1	1.16208 (11)	0.17662 (9)	0.45212 (6)	0.0500 (2)	
S2	1.14230 (12)	0.12822 (9)	0.22118 (7)	0.0539 (2)	
S3	0.71611 (11)	0.33291 (8)	0.15538 (7)	0.0519 (2)	
S4	0.72980 (11)	0.37770 (10)	0.43285 (6)	0.0561 (2)	
Si1	0.65327 (11)	0.67844 (9)	0.19121 (7)	0.04113 (19)	

### Atomic displacement parameters (Å^2^)

**Table:**

	*U*^11^	*U*^22^	*U*^33^	*U*^12^	*U*^13^	*U*^23^
C1	0.0479 (16)	0.0476 (16)	0.0509 (17)	0.0014 (13)	−0.0094 (13)	−0.0116 (13)
C2	0.0408 (14)	0.0361 (14)	0.0462 (15)	−0.0036 (11)	−0.0095 (11)	−0.0106 (12)
C3	0.0349 (13)	0.0416 (14)	0.0396 (14)	−0.0054 (11)	−0.0040 (11)	−0.0063 (12)
C4	0.0450 (15)	0.0410 (15)	0.0408 (15)	0.0001 (11)	−0.0060 (12)	−0.0085 (12)
C5	0.0355 (13)	0.0572 (17)	0.0579 (17)	−0.0006 (12)	0.0021 (12)	−0.0317 (14)
C6	0.0622 (19)	0.0425 (16)	0.087 (2)	−0.0083 (14)	0.0008 (17)	−0.0189 (16)
C7	0.0345 (14)	0.0643 (19)	0.078 (2)	0.0007 (13)	−0.0026 (14)	−0.0239 (17)
O1	0.0599 (13)	0.0760 (15)	0.0739 (15)	0.0266 (11)	−0.0219 (11)	−0.0261 (12)
S1	0.0450 (4)	0.0574 (4)	0.0450 (4)	0.0013 (3)	−0.0140 (3)	−0.0141 (3)
S2	0.0602 (4)	0.0494 (4)	0.0527 (4)	0.0113 (3)	−0.0155 (3)	−0.0214 (3)
S3	0.0549 (4)	0.0422 (4)	0.0610 (5)	−0.0007 (3)	−0.0273 (3)	−0.0176 (3)
S4	0.0457 (4)	0.0701 (5)	0.0429 (4)	0.0004 (3)	0.0075 (3)	−0.0114 (4)
Si1	0.0305 (3)	0.0406 (4)	0.0525 (4)	−0.0031 (3)	0.0015 (3)	−0.0171 (3)

### Geometric parameters (Å, º)

**Table:**

C1—O1	1.200 (3)	C5—S4	1.812 (3)
C1—S1	1.768 (3)	C5—Si1	1.865 (3)
C1—S2	1.767 (3)	C5—H5A	0.9700
C2—C3	1.336 (4)	C5—H5B	0.9700
C2—S2	1.747 (3)	C6—Si1	1.852 (3)
C2—S3	1.749 (3)	C6—H6A	0.9600
C3—S1	1.746 (3)	C6—H6B	0.9600
C3—S4	1.753 (3)	C6—H6C	0.9600
C4—S3	1.813 (3)	C7—Si1	1.853 (3)
C4—Si1	1.873 (3)	C7—H7A	0.9600
C4—H4A	0.9700	C7—H7B	0.9600
C4—H4B	0.9700	C7—H7C	0.9600
			
O1—C1—S1	125.6 (2)	Si1—C6—H6B	109.5
O1—C1—S2	122.9 (2)	H6A—C6—H6B	109.5
S1—C1—S2	111.47 (17)	Si1—C6—H6C	109.5
C3—C2—S2	116.77 (19)	H6A—C6—H6C	109.5
C3—C2—S3	127.3 (2)	H6B—C6—H6C	109.5
S2—C2—S3	115.90 (16)	Si1—C7—H7A	109.5
C2—C3—S1	117.2 (2)	Si1—C7—H7B	109.5
C2—C3—S4	126.9 (2)	H7A—C7—H7B	109.5
S1—C3—S4	115.92 (16)	Si1—C7—H7C	109.5
S3—C4—Si1	115.17 (15)	H7A—C7—H7C	109.5
S3—C4—H4A	108.5	H7B—C7—H7C	109.5
Si1—C4—H4A	108.5	C3—S1—C1	97.14 (15)
S3—C4—H4B	108.5	C2—S2—C1	97.32 (14)
Si1—C4—H4B	108.5	C2—S3—C4	100.85 (12)
H4A—C4—H4B	107.5	C3—S4—C5	102.02 (14)
S4—C5—Si1	116.21 (14)	C6—Si1—C7	112.72 (14)
S4—C5—H5A	108.2	C6—Si1—C5	107.85 (14)
Si1—C5—H5A	108.2	C7—Si1—C5	108.97 (14)
S4—C5—H5B	108.2	C6—Si1—C4	107.65 (15)
Si1—C5—H5B	108.2	C7—Si1—C4	107.94 (13)
H5A—C5—H5B	107.4	C5—Si1—C4	111.76 (14)
Si1—C6—H6A	109.5		

### Hydrogen-bond geometry (Å, º)

**Table:**

*D*—H···*A*	*D*—H	H···*A*	*D*···*A*	*D*—H···*A*
C5—H5*B*···S1^i^	0.97	2.89	3.673 (5)	138

Symmetry code: (i) −*x*+2, −*y*+1, −*z*+1.
